# An Autonomous Operation Path Planning Method for Wheat Planter Based on Improved Particle Swarm Algorithm

**DOI:** 10.3390/s25175468

**Published:** 2025-09-03

**Authors:** Shuangshuang Du, Yunjie Zhao, Yongqiang Tian, Taihong Zhang

**Affiliations:** 1 College of Computer and Information Engineering, Xinjiang Agricultural University, Urumqi 830052, China; 320233398@xjau.edu.cn (S.D.); tyq@xjau.edu.cn (Y.T.); 2 Engineering Research Center of Intelligent Agriculture, Ministry of Education, Urumqi 830052, China; 3 Xinjiang Agricultural Informatization Engineering Technology Research Center, Urumqi 830052, China

**Keywords:** path planning, particle swarm algorithm, wheat planting operations, full coverage

## Abstract

To address the issues of low efficiency, insufficient coverage, and high energy consumption in wheat sowing path planning for large-scale irregular farmland, this study proposes an improved hybrid particle swarm optimization algorithm (TLG-PSO) for autonomous operational path planning. Building upon the standard PSO, the proposed method introduces a Tent chaotic mapping initialization mechanism, a Logistic-based dynamic inertia weight adjustment strategy, and adaptive Gaussian perturbation optimization to achieve precise control of the agricultural machinery’s driving orientation angle. A comprehensive path planning model is constructed with the objectives of minimizing the effective operation path length, reducing turning frequency, and maximizing coverage rate. Furthermore, cubic Bézier curves are employed for path smoothing, effectively controlling path curvature and ensuring the safety and stability of agricultural operations. The simulation experiment results demonstrate that the TLG-PSO algorithm achieved exceptional full-coverage operation performance across four categories of typical test fields. Compared to conventional fixed-direction path planning strategies, the algorithm reduced average total path length by 6228 m, improved coverage rate by 1.31%, achieved average labor savings of 96.32%, and decreased energy consumption by 6.45%. In large-scale comprehensive testing encompassing 1–27 field plots, the proposed algorithm reduced average total path length by 8472 m (a 5.45% decrease) and achieved average energy savings of 44.21 kW (a 5.48% reduction rate). Comparative experiments with mainstream intelligent optimization algorithms, including GA, ACO, PSO, BreedPSO, and SecPSO, revealed that TLG-PSO reduced path length by 0.16%–0.74% and decreased energy consumption by 0.53%–2.47%. It is worth noting that for large-scale field operations spanning hundreds of acres, even an approximately 1% path reduction translates to substantial fuel and operational time savings, which holds significant practical implications for large-scale agricultural production. Furthermore, TLG-PSO demonstrated exceptional performance in terms of algorithm convergence speed and computational efficiency. The improved TLG-PSO algorithm provides a feasible and efficient solution for autonomous operation of large-scale agricultural machinery.

## 1. Introduction

The development of new high-quality productive forces in agriculture and the construction of smart agriculture represents the main directions for future agricultural growth. The deep integration of agriculture with modern information technology can effectively improve traditional farming methods, enabling high-quality, efficient, and high-performance production. With the widespread use of automatic navigation systems in smart agriculture [1], unmanned autonomous driving technology for farm machinery has emerged in agriculture. Autonomous operation path planning is now a key research focus in unmanned driving [2]. Based on efficient path planning algorithms for unmanned farm machinery, it is possible not only to optimize field operation quality and increase efficiency, but also to help address issues such as labor shortages and rising labor costs. However, existing agricultural machinery path planning methods still exhibit significant limitations when dealing with complex and irregular fields. On the one hand, although complex terrains can theoretically be covered through geometric decomposition or raster scanning, in large-scale field operations these approaches often lead to redundant paths, increased energy consumption, and excessive turning maneuvers. On the other hand, most current studies are based on idealized field assumptions and fail to fully account for practical constraints such as headland structures, the minimum turning radius of machinery, and operational swath width, making the resulting plans difficult to execute efficiently in real-world agricultural operations. For small-scale seeding operations, traditional path planning methods usually determine the main driving direction based on the longest edge of the field [3]. Due to topographic constraints, farmland in practice rarely conforms to regular rectangular geometries, and this complexity increases the difficulty of achieving full coverage with traditional path planning methods. Especially in irregular large-scale farmland, existing algorithms face issues such as high path overlap, frequent turns, and excessive energy use caused by local extremum traps or lack of diversity. Therefore, researching optimal driving direction methods for agricultural machinery seeding in irregular-shaped and large-scale farmland fields is crucial for improving operation quality and efficiency.

In the field of full-coverage path planning for intelligent agricultural machinery, extensive research has been conducted on farmland geometric modeling, agricultural machinery physical constraints, and the improvement of operational efficiency. Among these, geometry-based path planning methods have emerged as a key research focus. Hameed et al. [4] proposed a three-stage optimization strategy based on genetic algorithms, which sequentially optimizes the driving direction, trajectory sequence, and block ordering. This approach significantly reduces overlapping coverage and non-working travel distance. Jin and Tang [5] employed a divide-and-conquer geometric modeling method to optimize the path direction and area partitioning in irregular fields, effectively minimizing travel distance and operation time. Han et al. [6] treated the operational driving angle as a decision variable and determined the path direction adaptively based on field boundary features, thereby improving the overall straight-line path length. However, traditional geometry-based methods generally rely on idealized models and exhibit poor performance in real-world fields characterized by complex boundaries or irregular shapes. Most of these methods focus solely on minimizing path length, failing to comprehensively consider key indicators such as turning frequency, coverage rate, and energy consumption. As field complexity increases, the computational cost of such algorithms grows exponentially, making it difficult to meet the demands of real-time planning, thus significantly limiting their practical applicability.

Regarding the generation of agricultural turning trajectories, Sabelhaus et al. [7] developed seven types of maneuverable turns based on continuous curvature modeling, ensuring smooth transitions from zero to maximum curvature. Fennema [8] introduced a dynamic headland width model combined with multi-objective decision-making to enhance turning adaptability across various implement configurations. Although these methods improve path smoothness, they often involve computationally intensive curve fitting, insufficient modeling of kinematic constraints, and a lack of adaptability to different machinery types, thereby limiting their generalizability and engineering applicability.

To improve adaptability across various field shapes and operational modes, Jeon et al. [9] proposed a hybrid coverage path planning system that integrates internal boustrophedon and external spiral strategies, enhancing edge-case handling in autonomous navigation operations. Zhai et al. [10] developed a block-based nested path planning method to improve path continuity, while Kai et al. [11] employed an improved simulated annealing algorithm to optimize crop row layouts and achieve full coverage in complex terrains. However, these approaches tend to be highly specialized, relying heavily on specific field scenarios and operational assumptions, and lack a unified algorithmic framework, which restricts their scalability. In response, Gonzalo Mier et al. [12] introduced the open-source platform Fields2Cover, which integrates various path generation and optimization algorithms through a modular architecture, addressing core challenges such as headland optimization, swath generation, and smooth turning paths. This significantly enhances the generalizability and engineering practicality of coverage path planning.

In the domain of intelligent optimization algorithms, research has evolved from early applications of single algorithms to hybrid models and structural innovations. Classical algorithms such as Particle Swarm Optimization (PSO) [13], Genetic Algorithms (GA) [14], Ant Colony Optimization (ACO) [15], Simulated Annealing (SA) [16], and Whale Optimization Algorithm (WOA) [17] have demonstrated notable performance in small- and medium-scale path planning problems, owing to their global search capabilities and implementation simplicity. However, when applied to large-scale and complex farmlands, these algorithms often suffer from limited convergence accuracy and susceptibility to local optima, making it difficult to achieve simultaneous optimization of path length and energy consumption.

To address these limitations, researchers have proposed a variety of hybrid and enhanced algorithms. Syed et al. [18] developed the Guided Auto-wave Pulse Coupled Neural Network (GAPCNN), incorporating directional wave excitation and dynamic thresholds for faster obstacle-avoiding path search. Wang et al. [19] enhanced the Bat Algorithm (IBA) using Differential Evolution (DE) [20] to improve safety and smoothness in 3D path planning. Zhang et al. [21] combined the Grey Wolf Optimizer with adaptive convergence and weighting strategies to boost 3D trajectory optimization performance. Ye et al. [22] introduced a hybrid Artificial Bee Colony algorithm enhanced with genetic exploration strategies (HABC-GA) for navigating complex environments. Liu et al. [23] employed a multi-strategy enhanced dung beetle optimization (MI-DBO) algorithm to achieve full coverage in standard quadrilateral orchard fields. While hybrid algorithms exhibit improved performance, they also introduce increased complexity, require extensive parameter tuning, and often lack theoretical guidance for algorithm integration, which limits their interpretability and practical deployment.

Due to its simple structure, rapid convergence, and high compatibility with other techniques, the Particle Swarm Optimization (PSO) algorithm remains a core method in agricultural path planning. Garg et al. [24] incorporated genetic operations into PSO (PSO-GA) to enhance algorithmic stability. Yu et al. introduced simulated annealing to avoid premature convergence. Huang et al. [25] combined A* search with PSO to improve path accuracy and node efficiency. Xie and Huang [26] further proposed PSO variants incorporating cognitive mechanisms and cylindrical coordinate optimization strategies to improve path search in complex environments. In recent years, classical chaotic mapping techniques such as Tent mapping and Logistic mapping have also been widely applied to the improvement of swarm intelligence optimization algorithms. For instance, Liu et al. [27] proposed a Tent-mapping-based PSO for unmanned aerial vehicle coverage in dynamic user scenarios, which improved the coverage rate of the global optimal solution.

Existing studies have demonstrated that chaotic mappings such as Tent and Logistic can improve the global search capability and convergence performance of PSO to a certain extent [28,29]. However, these efforts have mainly focused on fields such as function optimization [30,31], image processing [32,33,34], and wireless sensor networks [35,36], with limited applications in autonomous path planning for agricultural machinery. In particular, research remains scarce for path optimization problems in large-scale irregular farmland environments. Moreover, although various chaotic mappings have been used in previous studies to improve PSO, most of these improvements are single-module improvements and lack coordination between multiple mechanisms.

In contrast, this study proposes a hybrid improvement strategy that integrates Tent mapping, Logistic-based weight adjustment, and Gaussian perturbation to determine the optimal driving direction for field operations. This approach enables more scientific, flexible, and efficient path planning, addressing key challenges in large-scale irregular farmland operations, such as low planning efficiency, insufficient coverage, and high energy consumption. By optimizing the driving direction, the proposed method minimizes operation strips, reduces overlap in coverage areas, decreases operation time and energy consumption, while ensuring the feasibility and stability of the generated paths.

## 2. Materials and Methods

### 2.1. Operational Modeling

#### 2.1.1. Farm Information Processing

**Figure 1:**
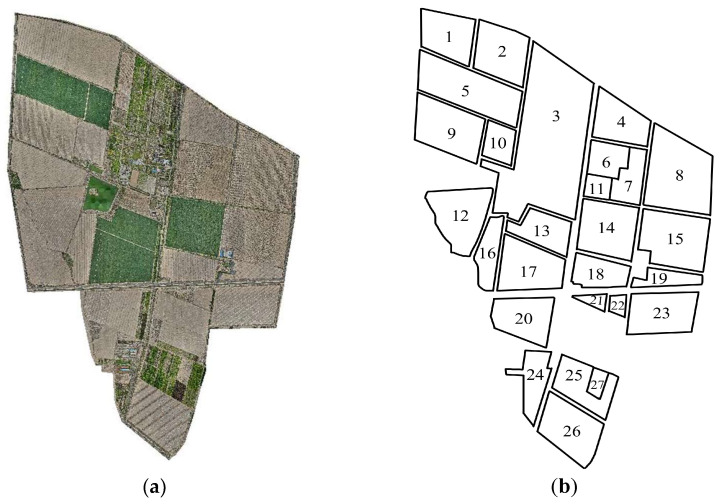
Satellite and outline maps of the farm. (**a**): Satellite map of the farm; (**b**): Outline of field.

This study selected Huaxing Farm in Xinjiang (44°13′ N, 87°17′ E) as the primary research area, employing a DJI Mavic 3 drone (DJI, Urumqi, China) as the data acquisition equipment to obtain spatial information data of the experimental farmland. The flight altitude was set at 110 m with a flight speed of 15 m/s, and the latitude and longitude coordinate data of the operational fields were ultimately stored in the appropriate format. To ensure precise field boundary accuracy, detailed annotations were made for the farm boundaries and individual field boundaries within the research area from the collected map data. The position coordinate system of the data acquisition equipment was WGS84 (World Geodetic System 1984). For the convenience of subsequent geometric modeling, the map data were converted to the UTM (Universal Transverse Mercator) coordinate system. This study develops a geospatial data processing algorithm for parcel data coordinate transformation based on geographical projection transformation methods. By constructing a spatial coordinate transformation model, the original longitude and latitude data of parcels are converted to planar projection coordinates. This achieves precise mapping from geographic coordinates to image coordinate systems, ultimately generating parcel boundary vector images with well-defined geometric characteristics. To further ensure experimental accuracy, the fields were sequentially coded from 1 to 27, with the experimental fields and their numbering shown in Figure 1.

#### 2.1.2. Farmland Environmental Modeling

**Figure 2:**
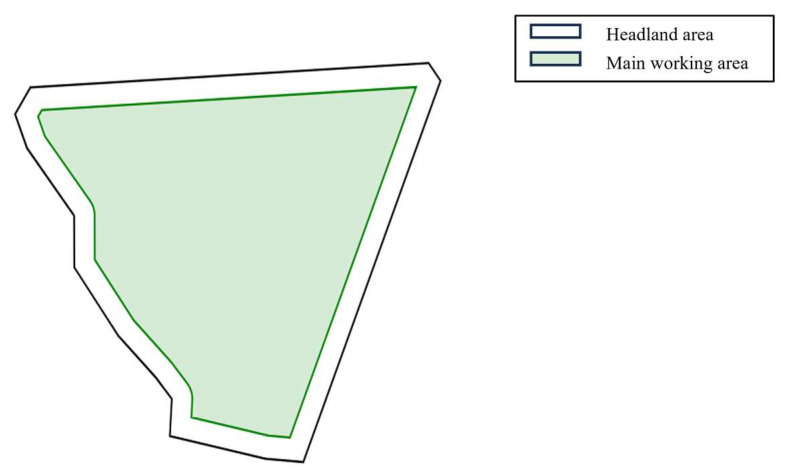
Geometry map method to build field simulation environment.

Based on the actual collected data information from each field, this paper employs the geometric graph method [37] to perform digital modeling and regional division of the farmland environment. The geometric graph method uses basic geometric elements such as points, lines, and surfaces to describe the shape and structure of individual fields, thereby achieving precise modeling of the operational space on irregular polygonal fields. In actual farmland operation path planning, to ensure the feasibility and efficiency of agricultural machinery operations, key regional divisions of the field must be conducted according to the operational characteristics of agricultural machinery, such as minimum turning radius and working width, including two parts: the headland area and the main working area. The headland area serves as a reserved buffer zone where agricultural machinery performs turning operations or temporary operational parking, and its width should meet the requirements of the turning radius. The main working area is the core cultivation zone of the field, where agricultural machinery conducts linear operations such as plowing, land preparation, seeding, and harvesting. The headland area width (W_h_) formula is shown in Equation (1):

(1) $$W_{h}=R\times |\cos\theta \;|+R+\frac{\omega }{2}$$

where R represents the minimum turning radius of the agricultural machinery (m), θ represents the angle between the current operation driving direction of the agricultural machinery and the x-axis (°), and ω represents the working width. In the modeling process, the headland width (W_h_) is calculated using Equation (1), and an inward buffer operation is employed to translate the field boundaries equidistantly inward, constructing the boundary lines between the headland area and the main working area to ensure sufficient turning space is reserved in the headland area within the geographical space. This division method establishes an accurate geometric foundation for subsequent agricultural machinery path planning and operation simulation. The simulation modeling results of field regional division are shown in Figure 2.

#### 2.1.3. Objective Function

This paper uses the main working area boundaries, working width, minimum turning radius of agricultural machinery, and other parameters as constraint conditions. Based on prior information such as the geometric shape of the working field, agricultural machinery parameters, and headland turning methods, a complete coverage path planning model for wheat seeding machines is constructed. According to different path optimization objectives, an improved particle swarm optimization algorithm is employed to search for the optimal operation driving direction of agricultural machinery within the 0–180° range, and then parallel scanning technology is utilized to generate operation strips parallel to the operation driving direction. A boustrophedon [38] back-and-forth walking path is used to cover the entire main working area. In the problem modeling stage, multiple potential optimization objectives were considered, including minimizing the effective operation path length, reducing the number of turns, and maximizing the coverage rate. Since the relative importance of these objectives varies across different application scenarios, this study ultimately adopts the minimization of the effective operation path length as the single optimization objective function. Meanwhile, the number of turns and coverage rate are employed as performance evaluation metrics to comprehensively assess the effectiveness of the algorithm. The three objective functions are formally defined as follows.

(1) Minimum total effective operation path length: During field operations, straight-line segments constitute the primary workload of agricultural machinery seeding operations, while turning segments between straight-line segments often involve non-operational movement, generating additional time consumption and energy consumption. To minimize the non-productive movement of agricultural machinery to the greatest extent, this paper defines the total effective operation path length as the sum of all straight-line operation segment lengths and uses this as the first optimization objective. Given a specific optimal operation driving direction angle θ, the agricultural machinery travels along several parallel operation rows within the main working area. The intersection points of the i-th operation row with the working area boundaries are sequentially marked as (x_i_^start^, y_i_^start^) and (x_i_^end^, y_i_^end^). The length of the i-th straight-line operation segment can be expressed as the Euclidean distance between these two intersection points, with the calculation formula shown in Equation (2). The sum of effective operation path lengths (L_s_) is calculated using the formula shown in Equation (3).

(2) $$d_{i}=\sqrt{{\left(x_{i}^{end}-x_{i}^{start}\right)}^{2}+{\left(y_{i}^{end}-y_{i}^{start}\right)}^{2}}$$

(3) $$L_{S}=\sum_{i=1}^{n}\sqrt{{\left(x_{i}^{end}-x_{i}^{start}\right)}^{2}+{\left(y_{i}^{end}-y_{i}^{start}\right)}^{2})}\;\;\;\;\;\;\;\;(i=1,2,...,n)$$

(2) Minimum number of turns: Turn path optimization is one of the key factors in improving the efficiency of agricultural machinery field operations. This study addresses the agricultural machinery operation path planning problem for convex polygonal fields and proposes an optimization method with the objective of minimizing the number of turns. When agricultural machinery performs field seeding operations according to the optimal operation driving direction, the total number of turns on the headland boundaries of polygonal fields should reach the minimum value. Optimization of the agricultural machinery turning frequency can not only reduce ineffective operation time but also decrease energy consumption and mechanical wear caused by frequent turning, which has important practical significance for improving modern agricultural operation efficiency. Let the polygonal experimental field be a convex polygonal field with m edges, where the length of the i-th edge is L_i_ (i = 1, 2,…, m), and the number of turns (N_i_) of agricultural machinery on the i-th edge of the polygonal field can be calculated using Equation (4).

(4) $$N_{i}=\frac{|\sin(\theta -\varphi_{i})|L_{i}}{2\omega }\;\;\;\;\;(i=1,2,...,m)$$

The total number of turns N of agricultural machinery on all boundaries of the polygonal field can be calculated using Equation (5).

(5) $$N=\sum_{i=1}^{m}N_{i}$$

(3) Highest path coverage rate: Path coverage rate is an important indicator for evaluating the quality of agricultural machinery field operations. Its core objective is to ensure that the working area is completely and uniformly covered while minimizing skip seeding and overlap seeding phenomena. Traditional serpentine or spiral path planning methods lead to uneven coverage in turning areas, with skip seeding problems particularly prone to occur at field boundaries. Therefore, this paper proposes a coverage control method based on optimal path planning, enabling agricultural machinery to efficiently cover the target area (F_w_) during autonomous operations. The coverage rate calculation formula is shown in Equation (6):

(6) $$F_{\mathrm{cov}}=\frac{F_{w}\cap \left\{U_{j}S^{j}\right\}}{F_{w}}$$

where F_cov_ represents the fraction of the main working area covered by operation strips, S^j^ represents the j-th operation strip, ∩ represents the intersection operator, and (∪_j_S^j^) is the union of all operation strips.

#### 2.1.4. Restrictive Condition

In autonomous agricultural machinery operation path planning, the reasonable setting of constraint conditions is the core to ensuring path feasibility, safety, and efficiency. Based on farmland geometric characteristics, agricultural machinery kinematic properties, and operational requirements, this study constructs the following multi-dimensional constraint system:

(1) Boundary constraints: The planned path must be strictly limited within the main working area of the field to avoid skip seeding or damage to farmland facilities caused by paths exceeding field boundaries. The main working area boundary is described by the vertex set A = {(x_1_,y_1_), (x_2_,y_2_),…, (x_n_,y_n_)}, and the ray casting method is employed for polygon collision detection. By calculating the intersection coordinates between operation strip endpoints and polygon boundaries, it is ensured that the starting and ending points of each operation path are always located within the main working area. For irregular polygonal fields, the Separating Axis Theorem (SAT) is introduced to optimize collision detection efficiency. SAT quickly determines the spatial relationship between paths and boundaries by calculating whether the projections of polygons on the separating axis overlap.

(2) Turning Radius Constraint: The minimum turning radius of agricultural machinery limits the curvature radius of the turning path, so the minimum turning radius (R) must be considered during path planning. This constraint can be addressed by reserving space through the derived headland width (W_h_), ensuring the feasibility of the machinery’s turning maneuvers. Different turning patterns impose varying requirements on the headland width. When the machinery adopts a bow-shaped turn, the turning radius (R) is less than half of the working width (ω). In the case of a semicircular turn, the turning radius (R) equals half of the working width ω. For bulb- or fishtail-shaped turns, the turning radius (R) must exceed half of the working width (ω).

(3) Path Continuity Constraint: During the sowing operation, the straight and turning paths of agricultural machinery must form a closed, continuous, and smooth trajectory to ensure sowing continuity and efficiency, avoiding issues such as reseeding, missed seeding, or mechanical damage caused by path discontinuities or abrupt curvature changes. High-precision GNSS (Global Navigation Satellite System) positioning and heading angle calibration are employed to ensure that the path connection error remains within a controllable range. The distance between the endpoints of adjacent operation strips must be smaller than the precision threshold (ε) of the positioning system, as expressed mathematically in Equation (7).

(7) $$∥(x_{i}^{end},y_{i}^{end})-(x_{i+1}^{start},y_{i+1}^{start})∥\leq \varepsilon$$

### 2.2. Full Coverage Path Planning

**Figure 3:**
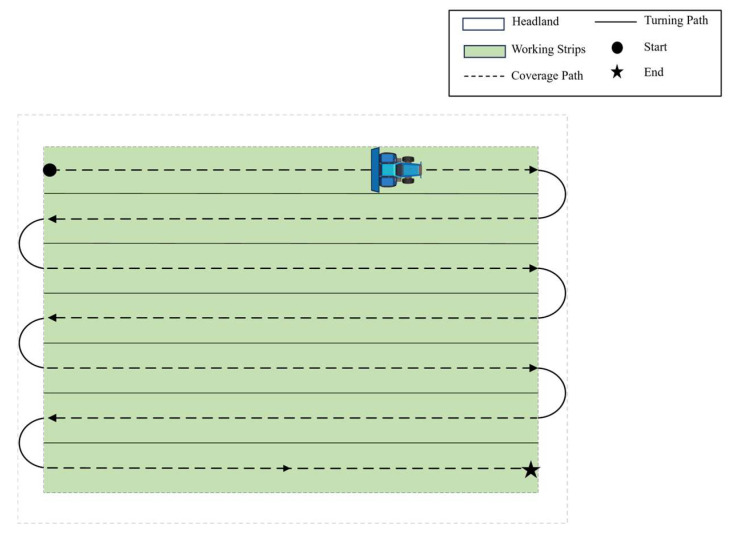
Schematic diagram of coverage path planning.

During the path planning for wheat sowing operations, the headland width (W_h_) is determined based on the minimum turning radius (R) of the agricultural machinery. Subsequently, the experimental field is divided into two distinct zones: the main working area and the headland area. The path planning process consists of several sub-modules, including headland generation, working swath generation, operational path generation, and turn path generation. In this study, an improved Particle Swarm Optimization (PSO) algorithm is employed to determine the optimal driving direction angle (θ) within the main working area, minimizing the effective operational path length. Based on the optimized angle, uniformly distributed working swaths are generated. The appropriate driving mode and turning pattern are selected according to the operational requirements and field characteristics, ultimately producing a complete coverage path. The full-coverage path planning result for a single field is illustrated in Figure 3.

#### 2.2.1. Mode of Operation

**Figure 4:**
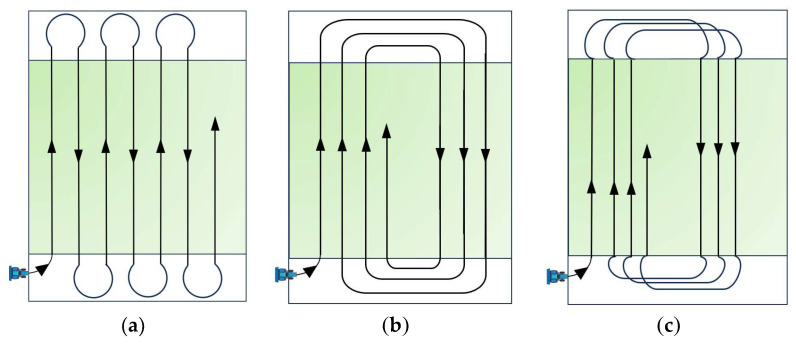
The common agricultural machine sowing methods. (**a**) Shuttle sowing; (**b**) centripetal sowing; (**c**) circuitous sowing. Schemes (**b**,**c**) are presented here as traditional examples, but due to their higher idle passes they are not adopted in the proposed method.

The working width and turning radius of a wheat seeder are typically closely related to its body width. Based on these characteristics, the full-coverage driving patterns of agricultural machinery can be classified. The most common methods for field sowing operations include shuttle sowing, centripetal sowing, and circuitous sowing [39], as illustrated in Figure 4.

Shuttle sowing, also known as back-and-forth sowing, involves straight reciprocating passes. The agricultural machinery travels in straight lines along a fixed direction, turns around in the headland area upon reaching the boundary, and then proceeds to the next adjacent swath until the entire field is sown. This method ensures high sowing quality, uniform machine wear, and simple field partitioning, while remaining unaffected by terrain variations. Centripetal sowing requires the machinery to enter the main working area from one side of the field. Although turning maneuvers are simpler, this method demands precise field division to avoid overlapping or missed sowing. Circuitous sowing involves a more complex driving pattern and is typically suited for shorter fields or ridge-based sowing operations. Similarly to centripetal sowing, it necessitates accurate field zoning to prevent reseeding or gaps in coverage.

#### 2.2.2. Vehicle Turning Strategies

**Figure 5:**
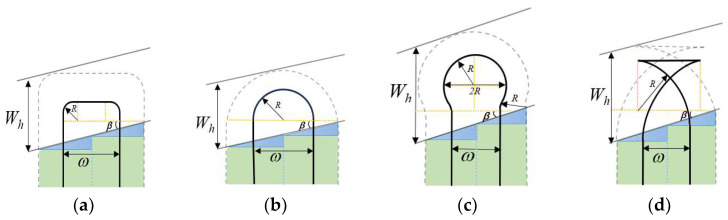
Common Farm Machinery Turning Styles. (**a**) Bow turning; (**b**) semicircular turning; (**c**) bulb turning; (**d**) fishtail turning.

In wheat seeding operations, the turning patterns of agricultural machinery exert a significant influence on operational efficiency, fuel consumption, and other performance indicators. Due to variations in field geometry, implement working width, turning radius, and other factors, the selection of appropriate turning patterns can minimize overlapping operations, reduce soil compaction, and enhance overall operational quality. Common tractor turning patterns primarily include arc turning, semi-circular turning, bulb turning, and fishtail turning, as illustrated in Figure 5.

When the minimum turning radius R of agricultural machinery is less than half the working width (ω/2), arc turning is selected (Figure 5a). When the turning radius R equals the working width ω, semi-circular turning is typically employed (Figure 5b). If the turning radius R is greater than half the working width, bulb turning (Figure 5c) or fishtail turning (Figure 5d) may be selected. The calculation formulas for the aforementioned turning strategies are as follows:

(8) $$L_{1}=2\omega \tan\beta +\pi R+\omega -2R$$

(9) $$L_{2}=2R\sin\beta +\pi R$$

(10) $$L_{3}=\omega \tan\beta +4R\arccos(R+\omega /2)/2R$$

(11) $$L_{4}=2R\tan\beta +\pi R+2R-\omega$$

### 2.3. Algorithm Description

Particle Swarm Optimization (PSO) is a global optimization algorithm based on swarm intelligence, originally proposed by Eberhart and Kennedy in 1995 [40], inspired by the collaborative mechanisms observed in bird flocking behavior during foraging. In the PSO algorithm, the optimization problem is abstracted as an optimal solution search task within a D-dimensional solution space. Each candidate solution is modeled as a particle, where the particle’s position represents the current solution and the particle’s velocity represents its movement tendency within the search space. The entire particle swarm consists of multiple particles, with each particle continuously adjusting its position and velocity by tracking its personal best position (p_best_) and the global best position (g_best_). Through this combination of local search and global collaborative mechanisms, particles iteratively converge toward the optimal solution within the solution space. The fundamental principles and procedures of the PSO algorithm are as follows:

(1) Initialize the particle swarm. Randomly generate a population of particles, where each particle represents a potential solution in the solution space. Each particle possesses its own position and velocity.

(2) Evaluate particle fitness. Calculate the fitness of each particle, namely the corresponding objective function value, to assess its performance at the current position.

(3) Update personal best. For each particle, compare the particle’s fitness and designate the current position as the personal best position (p_best_).

(4) Update global best. Select the global best (g_best_) from the personal best positions of all particles.

(5) Update particle velocity and position. Each particle updates its velocity and position according to Equations (12) and (13):

(12) $$v_{id}^{t}=wv_{id}^{t-1}+c_{1}r_{1}(P_{best}^{t-1}-x_{id}^{t})+c_{2}r_{2}(P_{gbest}^{t-1}-x_{id}^{t})$$

(13) $$x_{id}^{t}=x_{id}^{t-1}+v_{id}^{t}$$

where w represents the inertia weight, used to balance the particle’s current movement tendency with new search directions; v and x denote the velocity and position of particle i at the t-th iteration, respectively; c_1_ and c_2_ are learning factors representing the degree to which particles learn from individual and collective experiences; P_best_ represents the local optimal individual position, P_gbest_ represents the global optimal individual position, and r_1_, r_2_ are random numbers between 0 and 1, ensuring search randomness and diversity.

(6) Update personal best and global best. Recalculate the fitness of each particle based on new positions and update the personal best and global best accordingly.

(7) Terminate iteration. If termination conditions are satisfied, such as reaching the maximum number of iterations, the algorithm terminates; otherwise, return to step five and continue updating particle velocities and positions.

### 2.4. Improvements to the PSO Algorithm

PSO algorithm guides particles to update their positions based on individual and collective historical optimal solutions by simulating swarm intelligence behavior, gradually approaching the optimal solution. In complex optimization problems such as agricultural machinery path planning, PSO algorithm has gained application due to its simple structure and relatively fast convergence. However, the standard PSO algorithm exhibits significant limitations: its global exploration capability is relatively weak, and when dealing with complex solution spaces or multiple local optima, it is prone to premature convergence and struggles to identify the global optimal path. To address these issues and optimize the path planning effectiveness for farmland linear seeding operations, this paper proposes an improved particle swarm optimization algorithm oriented toward operation-driven direction angle optimization based on the principles of traditional PSO. Targeting the optimization characteristics of angular parameters in continuous space, through collaborative optimization involving chaotic sequence initialization of particle populations, dynamic inertia weight adjustment, and hierarchical perturbation strategies, the algorithm achieves the objective of total path length minimization while enhancing solution accuracy, robustness, and convergence speed in irregular farmland path planning problems.

#### 2.4.1. Initialization Based on Tent Chaotic Mapping

In the path planning optimization process, the quality of the initial particle population distribution has a decisive impact on the overall performance of the algorithm. The initialization of the population not only affects the global exploration ability of the solution space but also directly influences the convergence speed and optimization accuracy of the algorithm. Traditional Particle Swarm Optimization (PSO) algorithms generally initialize particle positions randomly and uniformly. While this approach is simple to implement, it often leads to uneven particle distribution and insufficient population diversity when dealing with the optimization of operational angles. This increases the risk of falling into local optima and limits the global search ability of the algorithm.

Although chaotic initialization strategies have been applied in PSO improvement studies, existing methods mainly target general function optimization problems and lack design considerations specific to agricultural machinery path planning applications. Liu et al. [41] proposed an early particle swarm optimization algorithm incorporating chaotic search, leveraging the traversal and randomness of chaotic variables to help particles escape from local optima. Although Nie et al. [42] introduced Tent chaos into Cat Swarm Optimization (CSO) for maximum power point tracking in photovoltaic systems, their chaotic mapping was only used to improve the initial population distribution and did not consider the actual physical significance of parameters.

In this study, an improved Tent chaotic mapping initialization strategy is proposed. Based on the periodic characteristics of the farmland operational angle parameters in the [0°, 180°] interval, a small random disturbance term is introduced to prevent the mapping from converging to a fixed point, which is a shortcoming of the traditional Tent mapping. Furthermore, the Tent mapping is combined with farmland boundary constraints to ensure that the initial particles are not only uniformly distributed in mathematical terms but also conform to the physically feasible domain of actual operations. As a typical chaotic system, Tent mapping exhibits good traversal and uniform distribution properties, which can prevent the early population from falling into local optima, effectively extracting and capturing information in the solution space. The iterative calculation formula for Tent mapping is shown in Equation (14).

(14) $$X_{t+1}=\left\{\begin{array}{c} 2X_{t},\;\;\;\;\;\;\;\;\;\;\;\;\;\;0<X_{t}<0.5\\ 2(1-X_{t}),\;\;\;\;\;\;0.5\leq X_{t}\leq 1 \end{array}\right.$$

To prevent the mapping from falling into fixed points, a small random perturbation term δ∼U(−10^−4^, 10^−4^) is introduced to generate initial particle positions, ensuring sequence ergodicity. By mapping the chaotic sequence to the solution space θ ∈ [0°, 180°], the generated initial population maintains randomness while exhibiting more uniform spatial coverage characteristics. Compared to traditional mapping, the probability density function of Tent mapping more closely approximates uniform distribution, effectively reducing spatial correlation between adjacent particles and avoiding operation direction search blind spots.

#### 2.4.2. Improved Inertia Weight Based on Logistic

In Particle Swarm Optimization (PSO) algorithms, the inertia weight (w) is a key parameter that balances the global exploration and local exploitation capabilities. The traditional linear decreasing weight strategy has the issue of insufficient flexibility in adjustment: a large ω value enhances global exploration but may cause oscillation in the particle trajectory, which affects convergence efficiency; whereas a small ω value weakens the exploration inertia of the particles, limiting their exploitation ability, and increasing the risk of getting trapped in local optima, thus hindering effective search in the solution space.

The DCWPSO [43] proposed by Han introduces a dynamic oscillating inertia weight, which improves the balance between exploration and exploitation. However, its oscillation pattern is preset and cannot adaptively adjust based on the actual optimization process. Wang proposed a dynamic inertia weight adjustment strategy based on new feature selection, which is mainly used for SVM parameter optimization [44]. The weight adjustment in this method depends on the feature selection results and is not applicable to continuous-space path planning problems. For the multi-modal, non-convex objective function characteristics in the farmland linear operation path optimization problem, the fixed inertia weight strategy is ineffective in minimizing the total path length in complex terrains.

To address this issue, this study proposes a nonlinear inertia weight dynamic adjustment mechanism based on Logistic chaotic mapping. The search weight is dynamically adjusted during the iteration process, with a higher inertia weight retained initially to enhance global search capability, and a smaller search radius introduced later through chaotic disturbance to improve local convergence speed. Compared to the linear decreasing method, chaotic weight adjustment effectively improves the non-repetition of the search trajectory, thereby reducing the risk of getting trapped in local extrema. The definition of the Logistic mapping expression is shown in Equation (15):

(15) $$r_{t+1}=4r_{t}(1-r_{t})$$

(16) $$w_{t}=r_{t}w_{\min}+\frac{(w_{\max}-w_{\min})t}{T_{\max}}$$

where w_min_ = 0.5, w_max_ = 0.9; rt is the random number generated after t iterations of for Equation (15); and T is the maximum number of iterations.

#### 2.4.3. Gaussian Perturbation Strategy

To further enhance the local search capability of the algorithm in the later stages of optimization and alleviate the issues of particle swarm optimization (PSO) getting trapped in local optima or experiencing search stagnation during subsequent iterations, this study introduces an adaptive Gaussian perturbation mechanism based on the search process. Although existing research on Gaussian perturbation strategies has achieved certain results, their application remains relatively simplistic. The AGMPSO [45] adjusts the Gaussian perturbation amplitude using a cosine law, which enables adaptive perturbation adjustment. However, its perturbation only affects the global best particle, neglecting other potentially promising particles, thus limiting the exploration capacity of the algorithm. The ADGMPSO [46] proposed by Wang et al. designs a dimension-adaptive Gaussian mutation mechanism, which applies independent mutation intensities to the hyperparameters of different dimensions, enabling parallel optimization in a multi-dimensional discrete space. However, this strategy is designed for discrete hyperparameter spaces. In contrast, agricultural machinery path planning requires optimization in continuous angular spaces, where changes in operation angles directly affect the spatial relationships of adjacent operation strips and the overall continuity of the path. The dimension-adaptive strategy of ADGMPSO not only leads to wasted computational resources when dealing with such single continuous parameters, but more importantly, it neglects the spatial correlation and geometric constraints of the farmland operation path.

To address these limitations, this study designs a hierarchical perturbation mechanism, applying varying intensities of perturbation based on the particle’s fitness ranking. Smaller perturbations are applied to better particles for fine-tuned search, while larger perturbations are applied to poorer particles to promote exploration. Furthermore, a directional perturbation mechanism is introduced. Based on the farmland shape characteristics and the spatial distribution of the already planned path, perturbation intensity is increased in directions with a higher probability of improving solution quality, thus enhancing search efficiency. The perturbation intensity employs an adaptive decay strategy, where its standard deviation is inversely proportional to the number of iterations, ensuring global exploration capability in the early stages of the algorithm and local convergence accuracy in the later stages. This mechanism holds significant application value in linear operation path optimization. Due to the high sensitivity of angle parameters, even small adjustments in angles directly affect the total path length and coverage quality. The updated particle velocity update expression is shown in Equation (17):

(17) $$v_{id}^{t}=wv_{id}^{t-1}+c_{1}g_{id}^{t}(P_{best}^{t-1}-x_{id}^{t})+c_{2}g_{id}^{t}(p_{gbest}^{t-1}-x_{id}^{t})$$

where g^t^_id_ represents the Gaussian perturbation generated by particles during the t-th iteration. The Gaussian expression is shown in Equation (18).

(18) $$g_{id}^{t}=gaussian(\mu ,\sigma^{2})$$

**Figure 6:**
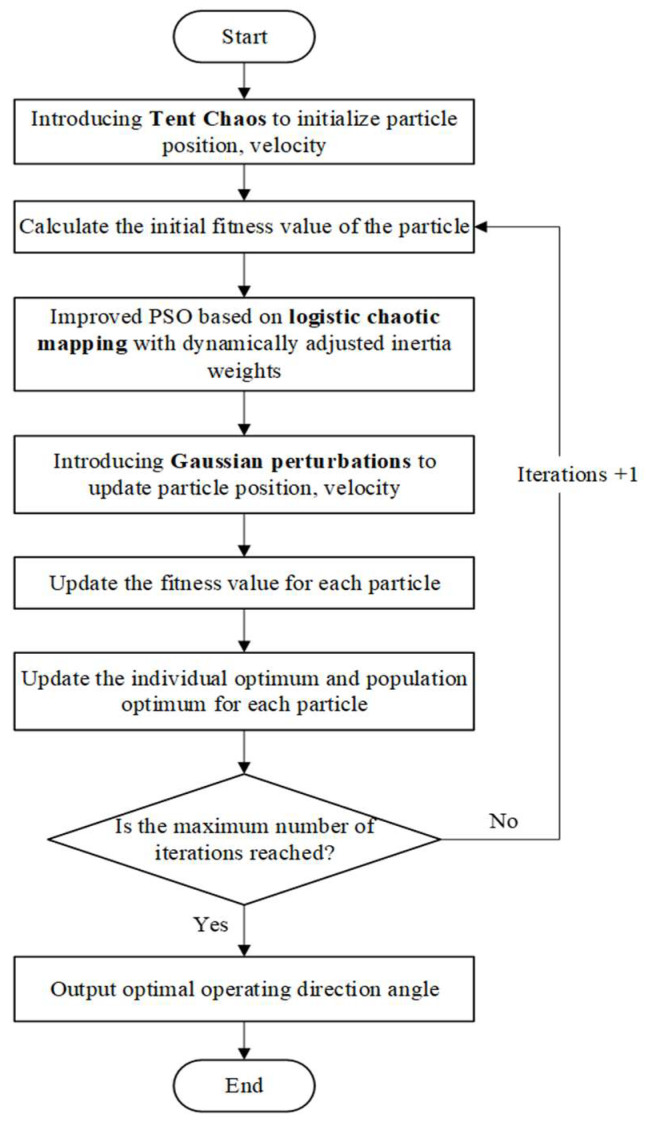
Flowchart of Improved Particle Swarm Optimization Algorithm.

In Equation (18), μ represents the standard deviation of Gaussian perturbation, and σ^2^ represents the variance of Gaussian perturbation. In this study, the particle swarm optimization (PSO) method is enhanced through the synergistic integration of three fundamental improvement strategies. The proposed TLG-PSO framework addresses the inherent limitations of conventional PSO methods by integrating these complementary enhancement mechanisms. The detailed implementation procedure of the algorithm is presented in pseudocode form in Algorithm 1. The corresponding flowchart of TLG-PSO is illustrated in Figure 6.
**Algorithm 1**. TLG-PSO.Initialize population and parameters num_particles = N MaxIter = T theta_min = 0° theta_max = 180° c1, c2 = learning factors Initialize chaotic variable r_0 and Gaussian parameters σ_0, σ_min # Phase 1: Tent chaotic initialization For i = 1:N do         Generate chaotic sequence u_i using Tent mapping (Equation (14))         Add small perturbation δ ~ U(−10^−4, 10^−4) to avoid fixed points         Map u_i into [theta_min, theta_max] to initialize position x(i)         Initialize velocity v(i) with small random values         Evaluate fitness Fit[i] = PathLength(x(i))         Set pbest[i] = x(i), fpbest[i] = Fit[i] End For Initialize gbest and fgbest from pbest # Phase 2: Iterative optimization (Logistic inertia and Gaussian perturbation) t = 0 While (t < T) do         Update chaotic variable r_t using Logistic mapping (Equation (15))         Compute dynamic inertia weight w_t using (Equation (16))         Compute adaptive Gaussian std σ_t = σ_0 × (1 − t/T) + σ_min         For i = 1:N do                 Generate random numbers r1, r2 ~ U(0, 1)                 Sample Gaussian perturbation g_ti using (Equation (18))                 Update velocity v(i) using (Equation (17)) with w_t and g_ti                 Update position x(i) = x(i) + v(i) with boundary handling                 Evaluate fitness NewFit[i] = PathLength(x(i))                 If NewFit[i] < fpbest[i] then update pbest[i] and fpbest[i]         End For         Update gbest and fgbest from current best particle         t = t + 1 End While Output the global optimal angle theta_best and the minimal path length L_best

## 3. Experiments and Results

### 3.1. TLG-PSO Algorithm Performance Test Experiments

#### 3.1.1. Test Functions

Four classic benchmark functions are selected for this study: Sphere, Ackley, Quartic, and Rastrigin. These benchmark functions provide a comprehensive means of testing and comparing the performance and efficiency of different algorithms when confronted with various types of optimization challenges. The specific mathematical expressions and characteristics of each function are presented in Table 1.

#### 3.1.2. Experimental Setup and Results

In this study, TLG-PSO is systematically compared with five other algorithms, Genetic Algorithm (GA), Ant Colony Optimization (ACO), Particle Swarm Optimization (PSO), Second-order Oscillatory Particle Swarm Optimization (SecPSO), and Hybrid Particle Swarm Optimization (BreedPSO), across four standard benchmark functions. The experiment uses Mean, Standard Deviation, and Optimal Value as the primary performance evaluation metrics to comprehensively assess the algorithms’ performance in terms of optimization accuracy, stability, and other factors.

In all test experiments, each algorithm was independently run 30 times to minimize the influence of random factors. The population size was uniformly set to 50, and the maximum number of iterations for a single run was 100. The experimental results are summarized in Table 2, reflecting the overall optimization capabilities of each algorithm across different function types.

**Figure 7:**
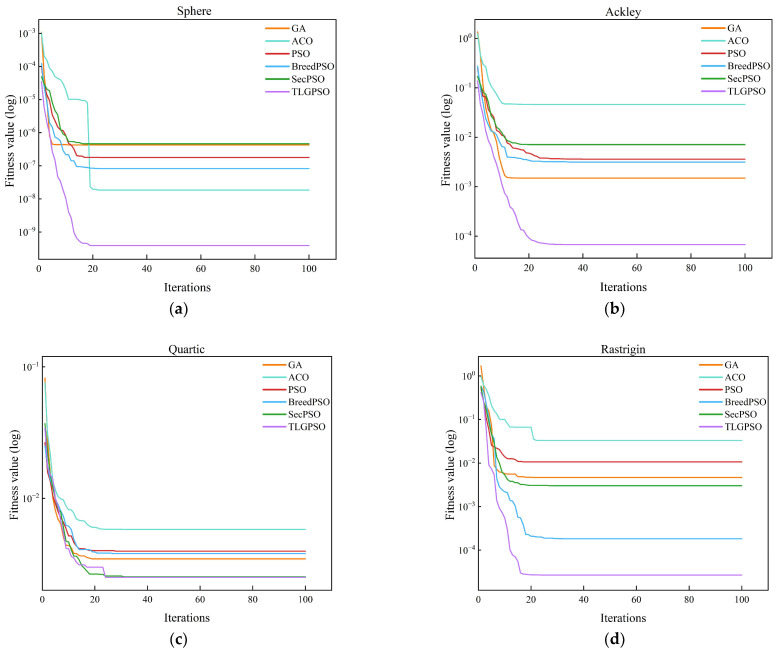
Average convergence curves of TLG-PSO versus GA, ACO, PSO, SecPSO and BreedPSO on four test functions. (**a**) Sphere function; (**b**) Ackley test function; (**c**) Quartic function; (**d**) Rastrigin function.

Based on the mean values, standard deviations, and optimal values of six algorithms tested on functions f1 through f4 as presented in Table 2, a comprehensive assessment of their overall performance in terms of solution quality and stability can be conducted. From the obtained mean and standard deviation results, it is observed that TLG-PSO achieves the lowest values for both metrics across all four test functions. An analysis of the convergence curves for different test functions shown in Figure 7 reveals that TLG-PSO demonstrates faster and more stable convergence behavior. In the Sphere unimodal function test, although GA obtains a lower optimal value, its mean and standard deviation are generally higher than those of other algorithms, indicating poorer stability.

In conclusion, it has been demonstrated that the TLG-PSO algorithm is capable of converging rapidly and efficiently to high-precision solutions when addressing simple optimization problems. When confronted with more complex multimodal functions that contain multiple local optima, the algorithm maintains excellent global search capabilities and successfully avoids becoming trapped in local optima. This comprehensive evaluation validates that TLG-PSO achieves a favorable balance between accuracy and stability, making it suitable for various optimization scenarios.

### 3.2. Path Planning Simulation Experiments

**Figure 8:**
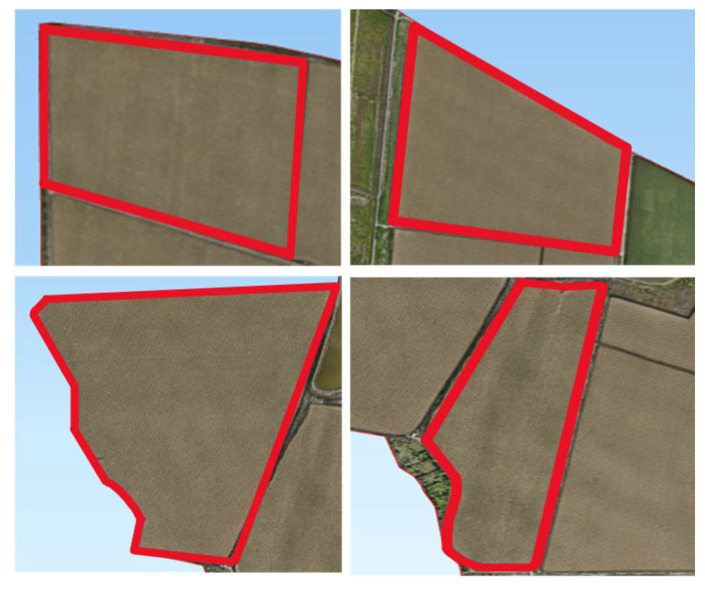
Four test fields selected based on shape complexity (Field 1, Field 4, Field 12, Field 16).

The simulation environment was constructed in this study based on field measurement data collected from Xinjiang Huaxing Farm. From the 27 agricultural fields at the experimental farm, four representative fields were selected according to terrain complexity, ranging from approximately regular to irregular geometries, to serve as research subjects for simulation experiments. The contour images of these four fields are presented in Figure 8, with specific field parameters detailed in Table 3. The experimental computational environment was configured as follows: Windows 11 (64-bit) operating system, Intel(R) Core(TM) i7-13620H processor, and 8 GB of RAM.

Significant variations in geometric structure and boundary morphology were exhibited by the selected field parcels, encompassing a diverse range of typical agricultural field configurations from regular to highly irregular forms. Among these, Field 1 was characterized by an approximately rectangular geometric structure with regular boundaries, resulting in relatively low path planning complexity. Field 4 was presented as an irregular trapezoid with boundaries composed of straight-line segments, demonstrating moderate geometric complexity suitable for conventional reciprocating operation patterns. Multiple convex and concave structures along with sharp corners were observed in the boundaries of Field 12, significantly increasing the difficulty of turning maneuvers in operational paths. Field 16 was similarly characterized by highly irregular boundaries with pronounced concave regions, where missed seeding and overlapping operations were likely to be induced under traditional operation modes. These representative field samples provided robust validation scenarios for investigating path planning methodologies under complex terrain conditions.

#### 3.2.1. Evaluation Metrics

In the study of wheat sowing operation path planning, the establishment of a scientific and reasonable evaluation index system is of great significance for optimizing operational efficiency and reducing production costs. This research proposes using labor savings rate and energy consumption reduction rate as core evaluation indicators. By quantitatively analyzing the efficiency of human resource allocation and the level of energy consumption in agricultural machinery operations, the actual effects of path planning algorithms in saving labor resources and reducing operational costs can be effectively assessed.

(1) Labor Savings Rate

The labor savings rate is a key indicator for measuring the efficiency of autonomous agricultural machinery operations. By comparing the differences in human labor input between traditional manual sowing methods and automated sowing operations, the level of automation in agricultural machinery operations can be objectively reflected. In real-world field operations, autonomous agricultural machinery typically operates at a preset constant speed during straight-line sowing and turning maneuvers, with higher speeds observed in straight-line segments compared to turning phases. The total time required to complete a sowing task in a given field generally comprises three components: the effective operation time along the sowing strips (T_work_), the turning time between adjacent paths (T_turn_), and the idle time (T_idle_) spent on auxiliary activities such as re-sowing, refilling, and refueling. Corresponding to these operational phases, the average number of laborers required is denoted as N_work_, N_turn_, and N_idle_, representing human resource demands for effective sowing, turning, and idle operations, respectively. In the case of fully autonomous machinery, it is reasonable to assume N = 0, indicating that no human intervention is needed throughout the operation. Therefore, the formula for total labor hours (T_l_) is shown in Equation (19).

(19) $$T_{l}=T_{work}N_{work}+T_{turn}N_{turn}+T_{idle}N_{idle}$$

The total number of labor hours used in traditional operations to complete a specific segment of operations in a field is T^h^, and the total number of labor hours used in autonomous operations is T^a^. The formula for calculating the labor saving rate (β) is as follows Equation (20).

(20) $$\beta =\frac{T^{h}-T^{a}}{T^{h}}\times 100\%$$

(2) Energy Consumption Reduction Rate

Reducing energy consumption in agricultural machinery and lowering farm production costs are of significant importance for farm operations. The energy consumption of agricultural machinery is not only closely related to its power and operation time but is also influenced by various factors, such as fuel consumption rate, mechanical efficiency, and path selection. In practical applications, improving the energy efficiency of agricultural machinery not only reduces energy expenditure but also minimizes its negative environmental impact. Optimizing the energy consumption of agricultural machinery through reasonable path planning is crucial for enhancing farm economic efficiency and sustainable development. The calculation formulas for agricultural machinery energy consumption (E) and energy consumption reduction rate (η) are given by Equations (21) and (22).

(21) $$E=P_{\mathrm{engine}}\times \left(T_{\mathrm{work}}+T_{\mathrm{turn}}\right)$$

(22) $$\eta =\frac{E_{b}-E_{a}}{E_{b}}\times 100\%$$

In these equations, P_engine_ represents the engine power of a specific model of agricultural machinery, E_b_ is the energy consumption before path optimization, and E_a_ is the energy consumption after path optimization.

In the simulation experiment, the straight-line cruising speed for autonomous agricultural machinery during sowing operations is set to 11 km/h, and the turning speed is set to 5 km/h. For traditional wheat sowing operations, each machine is operated by a driver, with the sowing speed of manually driven machinery set at 12 km/h and the turning speed at 6 km/h. Additionally, the idle time spent on operations such as refueling, entering the field, and replanting is uniformly set to 0.5 h. The engine power of the agricultural machinery is 55.93 kW/h.

#### 3.2.2. Comparative Experiments on Path Planning Methods for Fields of Different Shapes

To evaluate the adaptability and robustness of the proposed path planning method across different field geometries, this study selected four representative fields from the Huaxing Farm in Xinjiang—namely, Field 1, Field 4, Field 12, and Field 16—as experimental cases. These fields range from nearly regular rectangular shapes to highly irregular polygons, effectively capturing the diverse landform characteristics commonly encountered in agricultural production across the region.

A comparative experimental design was adopted, involving two path planning strategies:

(1) Traditional method: This approach generates operation strips based on the longest side of the field, with equal spacing between parallel strips. The path planning does not account for optimizing turning paths, resulting in straight-line paths along the field’s longest side. Although simple, this method fails to effectively reduce the operational inefficiencies and energy loss caused by turns. Particularly in fields with complex shapes, it may not achieve optimal results.

(2) The proposed improved method: In contrast, the improved method proposed in this paper uses Bézier curves to create smooth, semi-circular turns, thus reducing the power loss and efficiency decline associated with sharp turns. Additionally, curve transition sections are introduced at path junctions to enhance the continuity and smoothness of the operation, preventing instability due to sudden changes in direction.

The experiment is conducted in a unified simulation environment, both methods were implemented using the same working width constraint. The traditional method determined the operation direction based on the field’s longest edge, while the improved approach employed the proposed TLG-PSO to optimize the heading angle for path generation. Performance evaluation was conducted using several key metrics, including total path length, coverage rate, energy consumption, labor-saving rate, and energy reduction rate. These indicators comprehensively reflect the performance of the different path planning methods in practical operations, including aspects of operational efficiency, economic benefit, and adaptability. The experimental results are shown in Table 4, highlighting the relative effectiveness of the two methods under varying field conditions.

The experimental results demonstrate that the proposed TLG-PSO algorithm offers significant advantages in optimizing the operational heading angle. Compared to the conventional path planning strategy—which relies on the longest edge of the field as the reference for operation direction—the TLG-PSO approach yields notable improvements in both operational efficiency and quality. Through a comparative analysis of four typical fields, the optimized total path length was reduced by an average of 1557 m per field. Especially in fields 12 and 16, which have highly irregular geometries, the operational coverage rate increased to over 98.46%. In terms of energy savings, the TLG-PSO algorithm significantly reduced energy consumption during the operation process, with an average energy saving of 44.69 kW, and the labor-saving rate reached between 94.67% and 97.05%. This improvement in energy efficiency not only increased the economic benefits of agricultural production but also reduced the negative impact on the atmospheric environment. Particularly in field 16, the energy consumption reduction rate achieved by the TLG-PSO algorithm was as high as 12.75%. The TLG-PSO algorithm provides an effective solution for path planning in complex fields.

**Figure 9:**
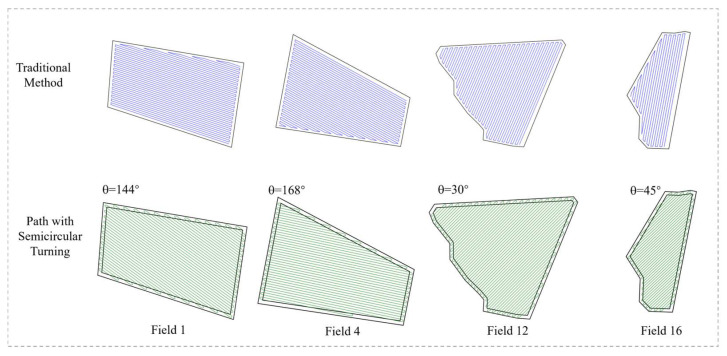
The full coverage path planning results using the traditional path planning method and our proposed semi-circular turn method with Bézier curve smoothing.

These findings clearly validate the effectiveness of the proposed algorithm in minimizing path length and reducing turning frequency. Its advantages are especially evident in complex and irregularly shaped fields, underscoring its adaptability and robustness. By determining the optimal heading direction through intelligent algorithmic optimization, the method not only enhances the efficiency of agricultural machinery operations but also contributes to a significant reduction in labor costs. Figure 9 presents visual comparisons of full-coverage operation paths generated by the two planning methods for the four typical fields (Field 1, Field 4, Field 12, and Field 16), which represent a gradual transition from near-regular to highly irregular shapes.

The traditional method and the proposed semicircular turning strategy with Bézier curve smoothing exhibit notable differences in path distribution, turning transitions, and trajectory smoothness. Compared to the conventional approach, the method presented in this study produces more compact and uniformly distributed operation paths, with smoother and more natural transitions at turning points. By optimizing turning trajectories based on a predetermined optimal heading angle using Bézier curves, the proposed approach effectively reduces the complexity of turning maneuvers and enhances the continuity and stability of the entire operational process.

It should be noted, however, that the experimental validation was conducted within a simulated environment using field boundary data obtained from UAV imagery. Real-world tests involving actual agricultural machinery have not yet been performed. As such, in practical applications, factors such as navigation errors, terrain variability, and operational uncertainties of agricultural equipment may affect performance. Therefore, further field trials are necessary to fully assess the adaptability and reliability of the proposed method under real agricultural working conditions.

#### 3.2.3. Comparative Experiments of TLG-PSO Algorithm in Path Optimization

**Figure 10:**
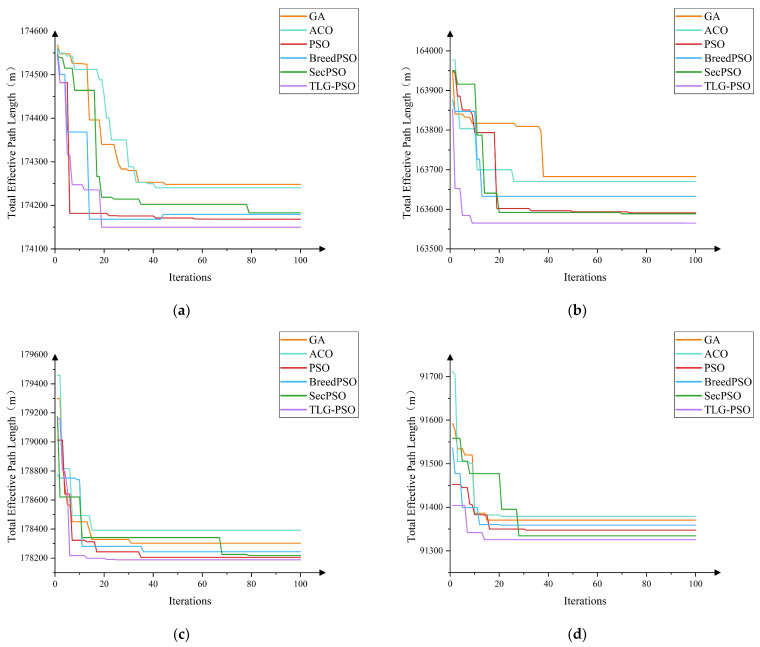
Comparison of the optimization process in different fields. (**a**) Field 1; (**b**) Field 4; (**c**) Field 12; (**d**) Field 16.

To validate the practical performance of the improved particle swarm optimization algorithm (TLG-PSO) in agricultural machinery path optimization, a comparative algorithm experiment was conducted with the objective of minimizing the effective operational path length. The experimental parameters for the agricultural machinery were set as follows: the working width *ω* was 5 m, and the minimum turning radius was 2.5 m. The parameters for TLG-PSO were configured as follows: population size *N* = 50 and maximum iteration count T = 100. The comparison algorithms included Genetic Algorithm (GA), Ant Colony Optimization (ACO), Particle Swarm Optimization (PSO), Hybrid Particle Swarm Optimization (BreedPSO), and Second-Order Oscillating Particle Swarm Optimization (SecPSO), with all algorithms maintaining consistent parameter settings. The convergence curves shown in Figure 10 display the optimization iteration processes of GA, ACO, PSO, BreedPSO, SecPSO, and TLG-PSO under four different fields. The path optimization results of TLG-PSO and the other five comparison algorithms across various fields are presented in Table 5.

During the population initialization phase, the TLG-PSO introduces the Tent chaotic map to enhance the uniform distribution of the population in the search space, effectively alleviating the problem of particle clustering that occurs during traditional random initialization. The initial paths generated by TLG-PSO on four irregular fields exhibit superior population diversity and initial search capability. An analysis of the convergence curves for the six algorithms in Figure 10 shows significant differences in convergence performance when the parameters are kept consistent. The GA and ACO algorithms exhibit slow convergence rates and insufficient global optimal solution exploration, resulting in longer path lengths compared to other algorithms. The PSO algorithm quickly reduces path length in the early iterations but is prone to stagnating at local optima. BreedPSO and SecPSO, two PSO-based variants, maintain relatively stable convergence trends during optimization; however, SecPSO converges too slowly to find the global optimum in a short period. In contrast, the proposed TLG-PSO algorithm is able to achieve faster convergence while finding the global optimal solution. As the number of iterations increases, the path length remains stable. In fields 12 and 16, which have complex shapes and winding boundaries, TLG-PSO, through the introduced adaptive Gaussian perturbation mechanism, dynamically adjusts the perturbation intensity based on the local search state, accelerating the global search and avoiding premature convergence to local optima. Within 20 generations, TLG-PSO demonstrates stable convergence, exhibiting stronger global search ability and optimization performance.

Table 5 presents the average performance results of TLG-PSO compared with five other algorithms across four field areas. The experimental results demonstrate that the TLG-PSO algorithm exhibits significant advantages in path optimization, achieving an average total operational path length of 154,620 m, which represents savings of 858 m and 756 m compared to ACO (155,478 m) and PSO (155,376 m), respectively. When addressing continuous optimization problems, ACO displays evident limitations in continuous space applications due to its discrete search mechanism. In cases where the solution space is continuous, the ACO algorithm requires discretization of continuous solutions to adapt to this type of problem, resulting in increased computational time consumption during the iterative process and further affecting overall operational efficiency.

The highest energy consumption indicator is observed in BreedPSO (823.71 kW), which represents an increase of 20.37 kW in energy consumption compared to TLG-PSO. Further analysis of the energy consumption indicators of the other five algorithms reveals that TLG-PSO achieves average energy consumption reduction rates ranging from 0.89% to 2.47%. Compared to other algorithms, TLG-PSO can effectively reduce energy consumption during the optimization process, thereby providing economic cost savings for agricultural production. Regarding algorithm convergence speed, SecPSO demonstrates poor performance with an average convergence speed of 64 generations. Although SecPSO introduces additional oscillation terms to enhance particle exploration capabilities, the experimental results indicate that this improvement actually constrains its convergence speed.

#### 3.2.4. Comprehensive Evaluation of Path Planning Optimization Methods Across All Fields

This study uses 27 fields from Huaxing Farm as experimental samples to evaluate the overall performance of the proposed TLG-PSO algorithm. The evaluation was performed across seven different methods, including the traditional path planning approach, GA, ACO, PSO, BreedPSO, SecPSO, and TLG-PSO. Four key performance metrics were used for assessment: average effective operational path length, average total path length, average coverage rate, and average energy consumption. The comprehensive experimental results of the seven methods across the 27 fields are presented in Table 6.

Analysis of the experimental results presented in Table 6 reveals that the TLG-PSO algorithm achieved an average shortest path length of 147,058 m. Compared to the traditional path planning method’s 155,530 m, an average reduction of 8472 m per field was observed, representing a 5.45% decrease. When benchmarked against other intelligent optimization algorithms, the TLG-PSO demonstrated an average improvement of 1098 m over GA (148,156 m) and 476 m over the second-best SecPSO algorithm (147,534 m). Although the improvement margins ranged between 0.32% and 0.74%, the cumulative path length savings across the 27 fields proved substantial, offering practical significance for operational efficiency enhancement.

Energy consumption performance was found to be highly correlated with path length. The average energy consumption of 762.73 kW achieved by the TLG-PSO algorithm was significantly lower than the 806.94 kW required by traditional methods, yielding an average energy savings of 5.48%. The TLG-PSO also consistently outperformed other intelligent algorithms, with average energy savings of 1.08% and 0.81% compared to GA and ACO, respectively. Regarding algorithmic efficiency, particularly notable performance was exhibited by the TLG-PSO algorithm. Convergence was achieved within an average of 20 iterations per field, surpassing all comparison algorithms except BreedPSO (21 iterations). A convergence speed improvement of 45.9% was particularly observed when compared to SecPSO (37 iterations). Furthermore, the optimization process for each field was completed by the TLG-PSO algorithm in an average of 19.53 s, representing a 42.9% reduction in computational time compared to the most time-consuming ACO algorithm (34.22 s).

Overall, superior performance was demonstrated by the TLG-PSO algorithm across all four key performance indicators when compared to both traditional path planning methods and other intelligent optimization algorithms. The significant reduction in computational time while maintaining solution quality positions this approach as particularly promising for real-time decision-making applications in large-scale agricultural field operations.

## 4. Discussion

As a key agricultural production region in China, Xinjiang has achieved a relatively high level of agricultural mechanization. However, the autonomous path planning of agricultural machinery in this region still faces significant challenges, primarily due to the large field sizes, complex boundaries, and irregular shapes that characterize much of the farmland. These conditions highlight the urgent need for efficient and intelligent path optimization methods to improve both operational efficiency and quality. To explore the core technologies of path planning in complex field environments, this study selects Fields 1, 4, 12, and 16 from Huaxing Farm in Xinjiang as representative samples to construct a robust and diverse dataset.

Simulation results show that the proposed path planning method consistently achieved superior full-coverage operation across all four field types. In particular, for complex fields such as Fields 12 and 16, the generated paths were more compact and logically distributed, with smooth boundary transitions and natural turning maneuvers. This led to improved continuity in the operational trajectory and more uniform coverage. Comparative analysis indicates that traditional path planning methods, constrained by fixed operation angles, often suffer from rigid path layouts in complex terrains, resulting in poor coverage performance, including blind spots and redundant passes, which severely compromise operational efficiency.

Despite the promising results, the proposed method still exhibits certain limitations. Specifically, when applied to fields with large-scale concave boundaries, the algorithm faces difficulties in generating rational subfield partitions. Achieving automated subdivision of complex fields into manageable subregions—without introducing redundant path segments—and coordinating localized path planning remains a key area for future research. Moreover, real-world factors such as navigation errors and the dynamic behavior of agricultural equipment must be further examined through field trials to fully assess their impact on path execution in actual farming environments.

## 5. Conclusions

This paper addresses the critical challenges associated with wheat seeding path planning in irregular farmland environments by proposing an improved Particle Swarm Optimization algorithm (TLG-PSO) that integrates Tent chaotic initialization, Logistic inertia weight dynamic adjustment, and adaptive Gaussian perturbation mechanisms. The algorithm introduces structural improvements to overcome the inherent deficiencies of standard PSO in complex path optimization, including premature convergence and insufficient search capability, thereby achieving comprehensive enhancement in intelligent path planning performance.

(1) Simulation experimental results demonstrate that TLG-PSO achieved superior full-coverage operation performance across all four categories of test fields. Compared to conventional fixed-direction path planning strategies, the proposed method exhibited significant advantages: average total path length was reduced by 6228 m, coverage rate increased by 1.31%, average labor savings reached 96.32%, and energy consumption decreased by 6.45%. In comparative analyses with GA, ACO, PSO, BreedPSO, and SecPSO algorithms, TLG-PSO achieved path length optimization ranging from 0.16% to 0.55% and energy consumption reduction from 0.96% to 2.47%.

(2) Large-scale field experiments further validated the algorithm’s practical value. In comprehensive testing encompassing 1–27 field plots, TLG-PSO demonstrated exceptional optimization capability. Compared to traditional methods, the average total path length optimization reached 5.45%, while average energy consumption decreased by 5.48%. In comparative experiments with other intelligent optimization algorithms including GA and ACO, path optimization ranged from 0.32% to 0.74%, with energy consumption reduction between 0.53% and 1.08%. Additionally, TLG-PSO exhibited superior performance in terms of convergence speed and computational efficiency.

Comprehensive analysis indicates that the TLG-PSO algorithm possesses robust optimization capability and engineering adaptability when addressing large-scale irregular convex field plots, achieving full coverage and smooth transitions in seeding paths with considerable practical application value. Future research should focus on in-depth exploration of concave field structure recognition and sub-field segmentation strategies, addressing how to automatically partition complex fields into manageable sub-plots without introducing redundant operational paths, thereby achieving coordinated integration of local paths.

## Figures and Tables

**Table 1:** Four test functions containing single and multiple peaks.

Function	Formula	Dimension	Domain	Optimal Value
Sphere	$f_{1}\left(x\right)=\sum_{i=1}^{d}x_{i}^{2}$	30	[−100,100]	0
Ackley	$f_{2}(x)=-20\exp(-0.2\sqrt{\frac{1}{d}\sum_{i=1}^{d}x_{i}^{2}})-\exp(\frac{1}{d}\sum_{i=1}^{d}\cos(2\pi x_{i}))+20+e$	30	[−32.8,32.8]	0
Quartic	$f_{3}(x)=\sum_{i=1}^{n}ix_{i}^{4}+rand(0,1)$	30	[−1.28,1.28]	0
Rastrigin	$f_{4}(x)=10d+\sum_{i=1}^{d}\left[x_{i}^{2}-10\cos(2\pi x_{i})\right]$	30	[−5.12,5.12]	0

**Table 2:** The performance of each algorithm was evaluated using Mean, Standard Deviation (Std) and Optimal Value over 30 independent runs. Bold font is used to emphasize values for the purpose of highlighting contrast.

Function	Evaluation Metrics	GA	ACO	PSO	BreedPSO	SecPSO	TLG-PSO
Sphere	Mean	4.26 × 10^−7^	1.84 × 10^−8^	1.78 × 10^−7^	8.24 × 10^−8^	4.62 × 10^−7^	3.89 × 10^−10^
	Std	2.03 × 10^−6^	3.69 × 10^−8^	2.92 × 10^−7^	1.83 × 10^−7^	1.01 × 10^−6^	7.01 × 10^−10^
	Optimal Value	2.24 × 10^−18^	5.67 × 10^−11^	7.20 × 10^−11^	1.16 × 10^−10^	1.38 × 10^−10^	1.63 × 10^−13^
Ackley	Mean	1.49 × 10^−3^	4.58 × 10^−2^	3.58 × 10^−3^	3.13 × 10^−3^	7.10 × 10^−3^	6.75 × 10^−5^
	Std	3.67 × 10^−3^	1.68 × 10^−1^	5.75 × 10^−3^	5.36 × 10^−3^	8.21 × 10^−3^	1.79 × 10^−4^
	Optimal Value	2.68 × 10^−9^	1.58 × 10^−5^	4.39 × 10^−5^	4.64 × 10^−6^	9.75 × 10^−5^	1.87 × 10^−12^
Quartic	Mean	3.46 × 10^−3^	5.80 × 10^−3^	3.96 × 10^−3^	3.80 × 10^−3^	2.53 × 10^−3^	2.50 × 10^−3^
	Std	3.34 × 10^−3^	6.12 × 10^−3^	3.57 × 10^−3^	3.47 × 10^−3^	2.87 × 10^−3^	2.86 × 10^−3^
	Optimal Value	4.40 × 10^−5^	4.57 × 10^−5^	3.77 × 10^−4^	5.71 × 10^−5^	3.73 × 10^−4^	1.72 × 10^−4^
Rastrigin	Mean	4.70 × 10^−3^	3.32 × 10^−2^	1.07 × 10^−2^	1.82 × 10^−4^	3.03 × 10^−3^	2.67 × 10^−5^
	Std	1.20 × 10^−2^	1.79 × 10^−1^	4.09 × 10^−2^	5.09 × 10^−4^	9.23 × 10^−3^	1.14 × 10^−4^
	Optimal Value	0	3.10 × 10^−9^	2.56 × 10^−9^	2.72 × 10^−10^	1.76 × 10^−7^	0

**Table 3:** Farmland parameters.

Field No.	Perimeter/m	Area/m^2^	Headland Width/m	Working Width/m	Turning Radius/m
1	2945.07	504,833	3	5	2.5
4	2742.38	447,559	3	5	2.5
12	2732.36	423,839.75	3	5	2.5
16	2438.3	357,550.71	3	5	2.5

**Table 4:** Results of full coverage path planning for different fields. The upward arrow (↑) indicates that a higher value of the indicator is preferred, while the downward arrow (↓) signifies that a lower value of the indicator is preferred.

Field No.	Path Length/m ↓		Coverage Rate/% ↑		Energy/kW ↓		Labour Savings Rate ↑	Energy Reduction Rate ↑
	Tra-Method	Improved Method	Tra-Method	Improved Method	Tra-Method	Improved Method		
1	178,702	176,642	99.40%	99.58%	934.11	913.35	96.82%	2.22%
4	172,317	166,605	99.12%	99.50%	902.71	865.66	96.72%	4.10%
12	190,968	181,308	96.19%	98.46%	1008.76	940.91	97.05%	6.73%
16	101,405	93,925	96.57%	98.97%	565.55	493.43	94.67%	12.75%

**Table 5:** Average performance comparison of TLG-PSO and five other algorithms across four fields. The downward arrow (↓) indicates that a lower value of the indicator is preferred.

Algorithm	Path Length/m ↓	Energy/kW ↓	Conv.speed (Iterations) ↓	Runtime/s ↓
GA	155,196	810.57	33	39.03
ACO	155,478	813.36	26	48.10
PSO	155,376	811.67	33	30.13
BreedPSO	155,039	823.71	20	29.84
SecPSO	154,874	811.13	64	22.74
TLG-PSO	154,620	803.34	17	21.05

**Table 6:** Comprehensive simulation results of five methods for full-coverage path planning in 27 fields. The downward arrow (↓) indicates that a lower value of the indicator is preferred.

Method	Path Length /m ↓	Energy/kW ↓	Conv.speed (Iterations) ↓	Runtime/s ↓
Traditional	155,530	806.94	−	−
GA	148,156	771.12	24	24.23
ACO	147,874	768.97	23	34.22
PSO	147,761	768.52	24	28.19
BreedPSO	147,675	767.71	21	25.76
SecPSO	147,534	766.78	37	24.43
TLG-PSO	147,058	762.73	20	19.53

## Data Availability

Data are available upon request.
